# Microstructure and Corrosion of Cast Magnesium Alloy ZK60 in NaCl Solution

**DOI:** 10.3390/ma13173833

**Published:** 2020-08-30

**Authors:** Zhen Li, Zeyin Peng, Kai Qi, Hui Li, Yubing Qiu, Xingpeng Guo

**Affiliations:** 1 School of Chemistry and Chemical Engineering, Huazhong University of Science and Technology, Wuhan 430074, China; fylz1989@hust.edu.cn (Z.L.); m201770328@hust.edu.cn (Z.P.); qikai@hust.edu.cn (K.Q.); 2 Key Laboratory of Material Chemistry for Energy Conversion and Storage, Huazhong University of Science and Technology, Ministry of Education, Wuhan 430074, China; 3 Changqing Oil and Gas Technology Institute, Changqing Oil Field Company, Xi’an 710021, China; lhui2_cq@petrochina.com.cn; 4 Hubei Key Laboratory of Materials Chemistry and Service Failure, Wuhan 430074, China; guoxp@mail.hust.edu.cn; 5 School of Chemistry and Chemical Engineering, Guangzhou University, Guangzhou 510006, China

**Keywords:** ZK60 magnesium alloys, microstructure, filiform-like corrosion, corrosion pit

## Abstract

In this work, the effects of the microstructure and phase constitution of cast magnesium alloy ZK60 (Mg-5.8Zn-0.57Zr, element concentration in wt.%) on the corrosion behavior in aqueous NaCl (0.1 mol dm^−3^) were investigated by weight-loss measurements, hydrogen evolution tests, and electrochemical techniques. The alloy was found to be composed of α-Mg matrix, with large second-phase particles of MgZn_2_ deposited along grain boundaries and a Zr-rich region in the central area of the grains. The large second-phase particles and the Zr-rich regions were more stable than the Mg matrix, resulting in a strong micro-galvanic effect. A filiform corrosion was found. It originated from the second-phase particles in the grain boundary regions in the early corrosion period. The filaments gradually occupied most areas of the alloy surface, and the general corrosion rate decreased significantly. Corrosion pits were developed under filaments. The pit growth rate decreased over time; however, it was about eight times larger than the general corrosion rate. A schematic model is presented to illustrate the corrosion mechanism.

## 1. Introduction

Magnesium (Mg) alloys have been widely applied as lightweight engineering materials due to their unique properties [1,2,3,4,5,6,7,8]. As commercial Mg-Zn-based alloys, ZK60 (Mg–Zn–Zr) alloys [9] have attracted great interest from researchers due to their high strength [10,11,12]. The microstructure [13,14], mechanical properties [15,16,17], and biological applications [18,19] of ZK60 alloys have been studied widely in recent decades. It has been verified that microstructure evolution is essential for the mechanical properties of ZK60 alloys, grain refinement, and stable precipitates, having vital effects on improving the mechanical properties [20,21,22,23]. Nevertheless, the weak corrosion resistance of ZK60 alloys limits their further applications.

The microstructure of Mg alloys, especially their second phases, has an evident impact on their corrosion behavior [3,5]. The second phases of Mg alloys may have a dual role in their corrosion, i.e., a galvanic acceleration effect or a corrosion blocking effect, depending on their quantities and distribution [24,25,26]. In Mg-Al alloys, when the amount of aluminum (Al) is low (e.g., Mg-5Al), the β-phase (Mg_12_Al_17_) is relatively discontinuous in the Mg matrix and mainly acts as a cathode phase to accelerate the dissolution of the matrix. As the content of the Al element increases (e.g., Mg-10Al), the β-phase precipitates are tiny and continuously distributed along the grain boundaries, producing a barrier to prevent corrosion [27]. The similar effect of the second phase was reported in other Mg alloys [28,29]. However, the effect of the second phase in ZK60 alloys on their corrosion behaviors is barely reported. Some researchers tried to enhance the corrosion resistance of ZK60 alloys by modifying their microstructure through heat treatment [30,31], deformation processing [32], and alloying [33,34,35,36]. Even so, the relationship between the microstructure and corrosion behavior of ZK60 alloys needs more studies for it to be investigated.

Some studies reported the corrosion behavior of ZK60 alloys on different occasions. Cheng et al. [37] pointed out that the Zr element in ZK60 alloys refined the grain and purified the alloy composition, which could improve its corrosion resistance in 1 M NaCl. Zeng et al. [38] investigated the effects of the microstructure and concentration of NaCl (3.5 and 5.0 wt.%) on the corrosion behavior of an extruded ZK60 alloy. They found that an increase in the grain size of the ZK60 alloy accelerated its corrosion rate. The alloy microstructure played a crucial role in the pitting and intergranular corrosion. Xu et al. [39] reported that the corrosion rate of a cast ZK60 alloy decreased with the immersion time in solutions containing 3.5 wt.% NaCl, NaBr, and NaI, while it displayed passivation in 3.5 wt.% NaF solution. Apart from the above reports, some studies focused on the biodegradation behavior of ZK60 alloys in Hank’s solution, Ringer’s solution, simulated body fluid, and artificial urine for biomedical applications [40,41,42,43]. The biodegradable property of the ZK60 alloys is the interesting issue in these investigations. In general, the above research usually concentrated on the uniform corrosion of ZK60 alloys, and little attention was paid to the development of their local corrosion. The influence of microstructure on the local corrosion of ZK60 alloys is still not clearly understood, especially the effect of the second phase.

In this study, a commercial cast ZK60 alloy was selected as the test material. Its microstructure was characterized by X-ray diffraction (XRD), scanning electron microscopy (SEM), energy-dispersive X-ray spectroscopy (EDX), and scanning Kelvin probe force microscopy (SKPFM) analysis. The general and local corrosion of the alloy in 0.1 M NaCl was investigated using weight loss tests, hydrogen evolution tests, and electrochemical measurements, as well as corrosion morphology monitoring with an optical microscope and SEM. The effect of the microstructure of the cast ZK60 alloy, especially the second phase and the distribution of alloying elements, on the corrosion initiation and developmental features of the alloy were investigated, and the mechanisms involved were studied. This work will help to verify the relationship between the microstructure and the corrosion behavior of ZK60 alloys. Moreover, it may also provide a theoretical basis for improving the corrosion resistance of ZK60 alloys by adjusting the microstructure in future research.

## 2. Materials and Methods

### 2.1. Test Material and Solution

A commercial as-cast ZK60 alloy was used in this study. Table 1 presents its chemical composition, analyzed by inductively coupled plasma-atomic emission spectrometry (ICP-AES, SPECTRO, Kleve, Germany). All the solutions used in this work were prepared with analytical-grade reagents and distilled water. The test solution was 0.1 M NaCl under an open-air condition, which was controlled at 25 ± 1 °C with a water bath.

### 2.2. Microstructure Characterization

The cast ZK60 samples (10 × 10 × 10 mm^3^) were sealed with epoxy resin (working area = 1.0 cm^2^), ground with 2000 grit SiC paper, and polished with 3 μm diamond paste. Then, they were etched with a picric acid solution for metallographic analysis. The metallographic structure of the cast ZK60 sample was observed using a 3D optical microscope (VHX-1000, KEYENCE, Osaka, Japan), SEM (Quanta 200, FEI, Eindhoven, The Netherlands) equipped with EDX (EDAX-Genesis), and transmission electron microscopy (TEM). The TEM sample was firstly mechanically ground to a thickness of about 20 μm, and then, it was ion milled at 4 keV and 4°, cooled by liquid nitrogen. TEM observations were carried out with an FEI Talos F200X transmission electron microscope (FEI, Portland, OR, USA) operated at 200 kV. The element content and elemental distribution of the cast ZK60 sample were characterized by EDX and an electron probe micro-analyzer (EPMA-8050G, SHIMADZU, Kyoto, Japan), respectively. Phase structure analysis was performed by XRD (X’Pert PRO, PANalytical B.V., Almelo, The Netherlands) using Cu Kα radiation. The scan range of 2θ was from 20° to 90° with a scan step of 0.02°. The XRD pattern was analyzed with the X’Pert HighScore Plus software (2.0, PANalytical B.V., Almelo, The Netherlands). SKPFM (SPM-9700, SHIMADZU, Kyoto, Japan) was used to measure the relative Volta potential differences among different microstructural constituents to show their relative nobility. Meanwhile, the corresponding topography maps of the same area were also obtained.

### 2.3. Electrochemical Tests

All the electrochemical tests were carried out using a CS 350 Corrtest electrochemical workstation (Wuhan Corrtest, Wuhan, China). The working electrode (10 × 10 × 10 mm^3^) was sealed with epoxy resin (working area = 1.0 cm^2^), which was ground with 2000 grit SiC paper and rinsed in distilled water and ethanol. A saturated calomel electrode (SCE) and a platinum (Pt) electrode were used as the reference electrode and the counter electrode, respectively. Polarisation curves were generated and electrochemical impedance spectroscopy (EIS) was performed, respectively, at different corrosion times. At free corrosion potentials (E_corr_), the EIS tests were performed with an AC voltage amplitude of 10 mV in the frequency range of 100 kHz–0.05 Hz. The EIS results were fitted with the Zview2.0 software. The polarization curves were generated at the scan rate of 0.5 mV s^−1^, scanning towards the positive direction. The corrosion current density (*i*_corr_, mA cm^−2^) was estimated by the cathodic Tafel extrapolation method, according to [5,44]. The corresponding corrosion rate (*P*_i_, mm y^−1^) was converted by the equation [5,45]:

(1) $$P_{i}=22.06i_{\mathrm{corr}}$$

where the corrosion current density *i*_corr_ is estimated by the Tafel extrapolation of the cathodic branch of the polarization curves, and *P*_i_ is related to the average corrosion rate.

All the electrochemical tests were repeated at least three times in this study.

### 2.4. Weight Loss Tests

The cast ZK60 specimens (20 × 20 × 4 mm^3^) were ground with 2000 grit SiC paper, rinsed in distilled water and ethanol, dried with cold air, and kept in a vacuum desiccator before the weight loss test. The specimens were corroded in 0.1 M NaCl for different times and then immersed in a CrO_3_ solution (180 g L^−1^, ~25 °C) for 10 min to remove corrosion products. At least three parallel tests were performed under each test condition. The average weight loss rate of the cast ZK60 alloy (Δ*W*, mg cm^−2^ h^−1^) can be converted to a general corrosion rate P_w_ (mm y^−1^) using [5,44]:

(2) $$P_{w}=3.6524\Delta W/\rho$$

where *ρ* is the metal density (g cm^−3^). For the cast ZK60 alloy, *ρ* = 1.8 g cm^−3^; thus, Equation (2) becomes:

(3) $$P_{w}=48.67\Delta W$$

### 2.5. Hydrogen Evolution Tests

**Figure 1:**
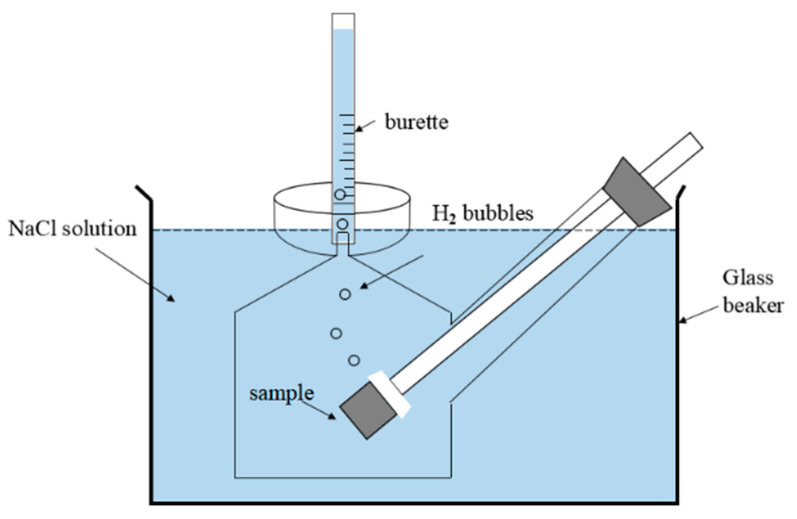
Schematic diagram of the test system for the hydrogen evolution measurement.

Following the work of Shi [46], plug-in specimens were employed to perform the hydrogen evolution test in 0.1 M NaCl at room temperature (~25 °C). Figure 1 presents the schematics of the test system. The cast ZK60 specimens (10 × 10 × 10 mm^3^) were treated as the above weight loss samples. The evolved hydrogen was collected into a burette, and its volume (*V*_H_, mL cm^−2^) was recorded at different times. The hydrogen evolution rate, v_H_ (mL·cm^−2^ day^−1^), can also be converted to a general corrosion rate, *P*_H_ (mm y^−1^), using [5,44].

(4) $$P_{H}=2.2\;v_{H}$$

### 2.6. Corrosion Morphology Characterization

The etched metallographic specimens were placed in 0.1 M NaCl, where the solution thickness on the sample surface was about 1 mm, to observe the corrosion development in the initial period (0–1 h) in situ using a 3D optical microscope (KEYENCE VHX-1000). The corrosion morphologies of the cast ZK60 samples corroded in 0.1 M NaCl for different times were measured by SEM and with the 3D optical microscope. The cross-section corrosion morphologies and the elemental distributions were analyzed by EPMA (EPMA-8050G).

## 3. Results and Discussion

### 3.1. Microstructure of the Cast ZK60 Alloy

**Figure 2:**
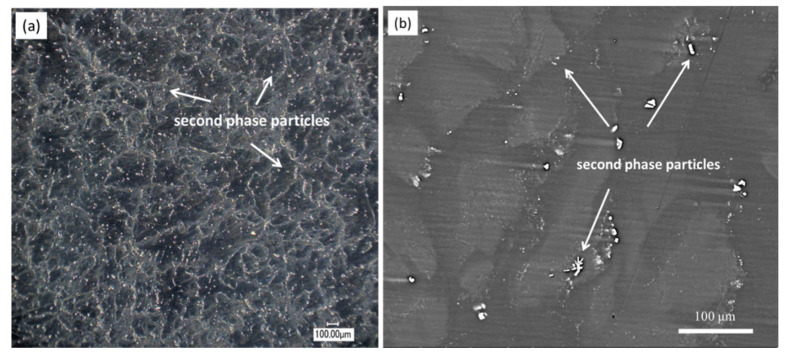
(**a**) Optical and (**b**) BSE-SEM micrographs of the cast ZK60 alloy.

**Figure 3:**
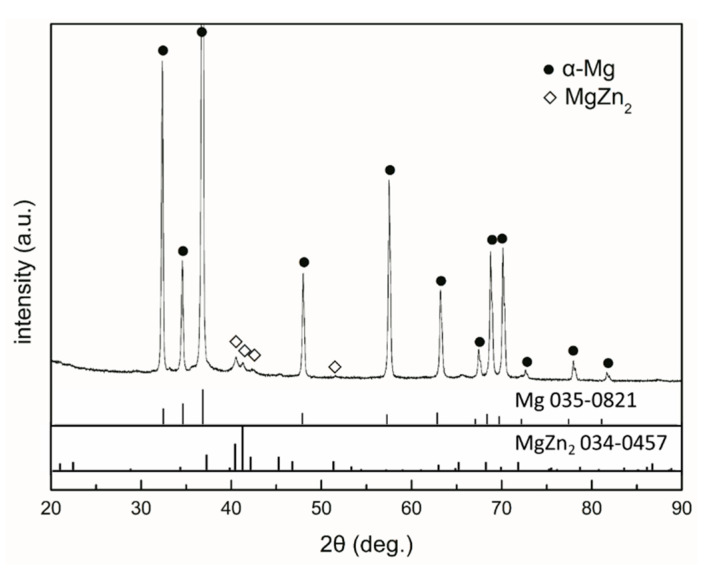
XRD pattern of the cast ZK60 alloy.

Figure 2 shows the optical and the back-scattered electron (BSE) SEM micrographs of the cast ZK60 alloy. The microstructure of the cast ZK60 alloy was composed of an α-Mg phase and large second-phase particles, which were mainly deposited along the grain boundaries (Figure 2a,b). According to [47,48], the main components of these particles are Mg and Zn and may be MgZn_2_ and MgZn. The XRD pattern of the cast ZK60 alloy in Figure 3 only displays the presence of MgZn_2_. No discernable diffraction peaks from MgZn were detected in this work, which suggests no MgZn phase in the studied alloy; also, there is no Zr detected, maybe due to its low content in the test sample. However, the XRD patterns cannot give accurate structural information for the second phase. Therefore, the crystal structures of the abovementioned second phases were characterized using TEM in detail as follows.

**Figure 4:**
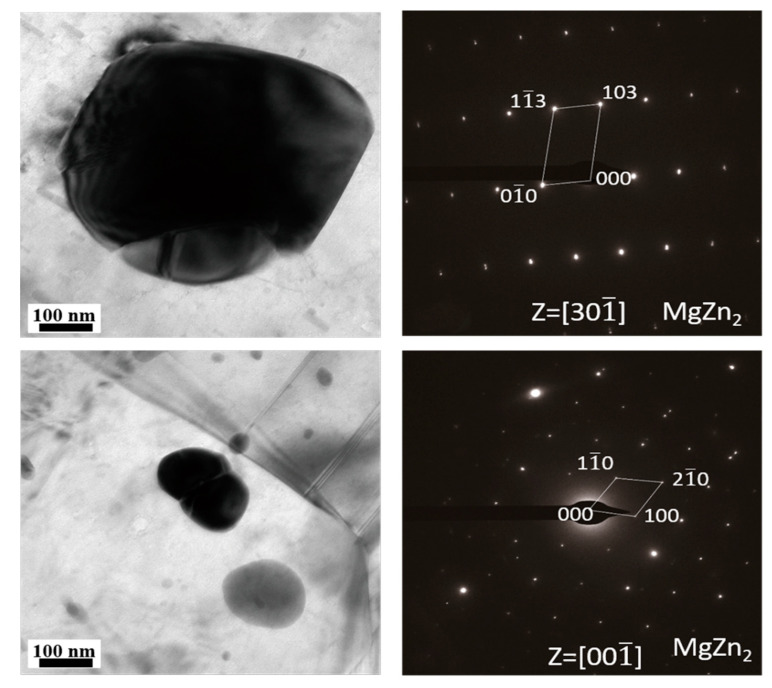
TEM micrograph of the cast ZK60 alloy and the corresponding selected area electron diffraction (SAED) patterns of the second-phase particles.

To further verify the second phase in the as-cast ZK60 alloy, Figure 4 presents the TEM micrograph of the as-cast ZK60 alloy. Block-shaped and globular second-phase particles can be observed in Figure 4. The blocky phase has a size of about 500 nm, and the globular phase is about 100 nm. Figure 4 also presents the corresponding selected area electron diffraction (SAED) patterns of the second phase. The SAED patterns proved that the two second-phase particles were MgZn_2_ [49]. No MgZn phase was found in the as-cast ZK60 alloy.

**Figure 5:**
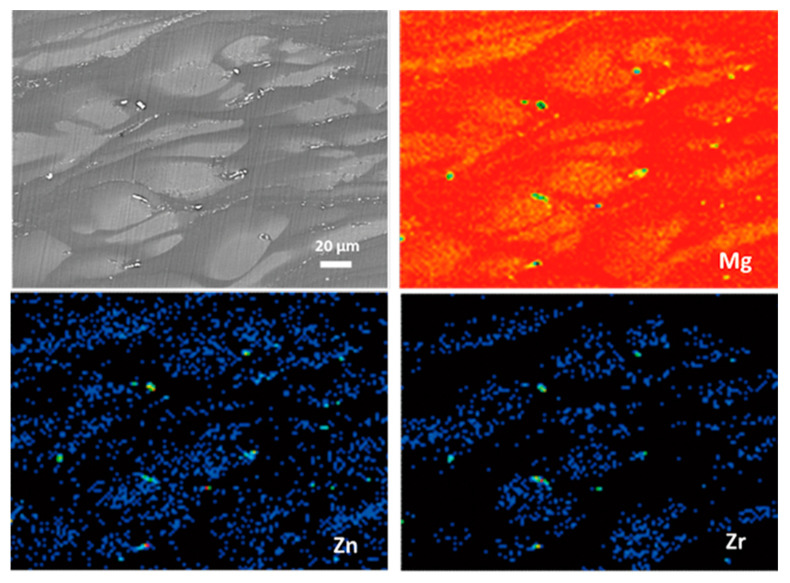
BSE-SEM image of the cast ZK60 alloy and the corresponding area distributions of Mg, Zn, and Zr.

The BSE-SEM micrograph in Figure 2b indicates the nonuniform distribution of the alloying elements in the cast ZK60 because the brighter areas contain more elements of higher atomic weight than the darker regions [50]. Figure 5 presents the area distribution of the alloying elements in the cast ZK60, proving that the center area of the grains with light color was richer in Zr and Zn (Zr-rich region) than the neighboring darker zones (grain boundary region). Here, the “grain boundary region” of the as-cast ZK60 is denoted as the areas between the Zr-rich region, i.e., the dark areas in Figure 2b. This uneven distribution of the alloying elements in the cast ZK60 alloy may cause the inhomogeneous electrochemical activity resulting in the micro-galvanic corrosion [51].

**Figure 6:**
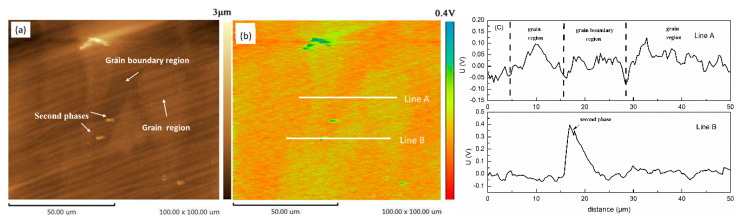
(**a**) Scanning Kelvin probe force microscopy (SKPFM) topography map; (**b**) Potential map of the same area; (**c**) Volta potential profiles along lines A and B in the SKPFM image.

Figure 6 presents the SKPFM maps of the cast ZK60 sample, which clearly show that the second phase had the highest potential. Furthermore, the Volta potential profiles (Figure 6c) along the line A and line B indicated that the central region of the grains (i.e., Zr-rich region) exhibited higher potential than those of the grain boundary regions, owing to the higher Zr and Zn contents in the center of grains and higher Mg content in the grain boundary regions (Figure 5). Thus, the second-phase particles and the central region of the grains should be more stable than the Mg matrix in grain boundary regions and more likely to become cathodes in the micro-galvanic cells. A second-phase particle occurred in the grain area (Figure 6a), which is consistent with Figure 2a, so the micro-galvanic corrosion may also have been initiated in the grains.

### 3.2. Weight Loss Tests

**Figure 7:**
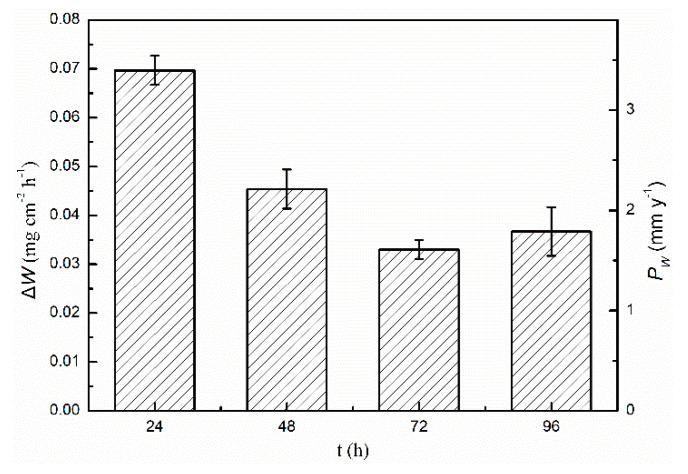
Corrosion rates (Δ*W* and *P*_W_) of the cast ZK60 alloy after immersion in 0.1M NaCl for different times measured by weight loss tests.

Figure 7 shows the Δ*W* (mg cm^−2^ h^−1^) and *P*_w_ (mm y^−1^) values of the cast ZK60 alloy immersed in 0.1 M NaCl for different times (t). The *P*_w_ at 24 h was the largest (3.5 ± 0.1 mm y^−1^), which is similar to that of a cast ZK60 in 0.9% NaCl (4.6 ± 0.6 mm y^−1^) reported by Merson et al. [52]. Then, it gradually decreased by approximately half to a relatively stable value (1.4~1.7 mm y^−1^) over 72–96 h. The decrease in *P*_w_ over 72–96 h may have been related to the increase in the corrosion product layer Mg(OH)_2_ on its surface [53].

### 3.3. Hydrogen Evolution Tests

**Figure 8:**
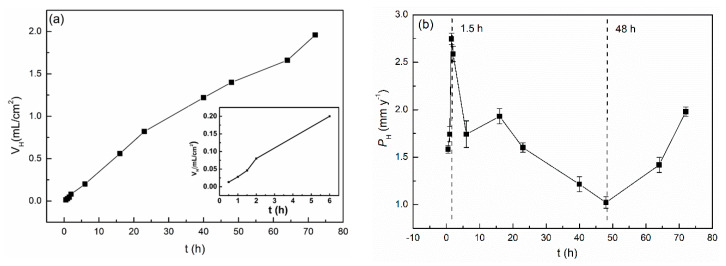
Changes in (**a**) *V*_H_ and (**b**) *P*_H_ of the cast ZK60 alloy as a function of time measured by the hydrogen evolution test in 0.1M NaCl.

Figure 8 presents the hydrogen evolution test results for the cast ZK60 alloy to show the change in its corrosion rate in detail, in which *v*_H_ is the differentiation of the *V*_H_-t curve in Figure 8a and *P*_H_ is calculated by Equation (4). The *V*_H_ at 24 h (Figure 8a) was about 0.7 ± 0.03 mL cm^−2^, which is higher than that of an extruded ZK60 (~0.5 mL cm^−2^) [54]. The *P*_H_ value (Figure 8b) increased with time in the initial corrosion period (0–1.5 h) and then decreased over 1.5–48 h; at last, it increased again over 48–72 h. These results imply that there exist different corrosion stages in the periods of 0–2 h, 2–48 h, and 48–72 h, which may be related to the change in the alloy surface condition. According to Song [24], the total volume of hydrogen collected should equal the total amount of metal lost, and both the weight loss and hydrogen evolution tests were reliable methods. Even though the hydrogen evolution rate *P*_H_ we measured is slightly lower than the weight loss corrosion rate *P*_w_, which may be caused by the difference in the test methods, they generally show similar change tendencies.

### 3.4. Polarisation Curve Measurements

**Figure 9:**
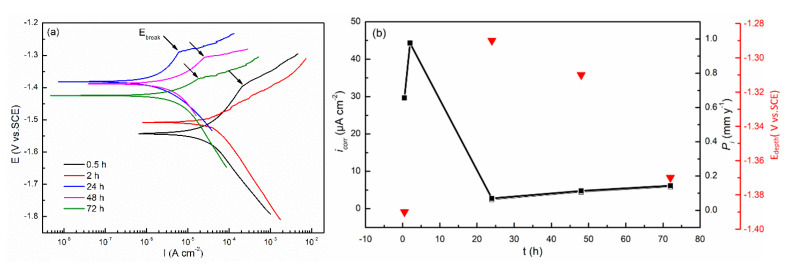
(**a**) Polarization curves of the cast ZK60 alloy immersed in 0.1 M NaCl for different times; (**b**) Changes in the i_corr_, P_i_, and E_break_ with time (25 °C).

Figure 9 presents the polarization curves of the cast ZK60 alloy after immersion in 0.1 M NaCl for different times and the changes in its corrosion rates (i_corr_ and P_i_) with time. All the polarization curves showed the typical features of activation-controlled processes [55,56]. A so-called breakdown potential (E_break_) occurred in the anodic polarization curves (except t = 2 h), owing to the breakdown of the oxide film on the alloy [57], as shown in Figure 9b. E_corr_ (Figure 9a) and E_break_ (Figure 9) moved positively in the period of 0–24 h and then became negative again, while P_i_ and i_corr_ (Figure 9b) displayed a similar change with corrosion time to that of P_H_ as shown in Figure 8b, which is consistent with previous reports [38,58]. The i_corr_ and P_i_ values in Figure 9b are much smaller than the P_w_ (Figure 7) and P_H_ (Figure 8b) values. Similar results from other Mg alloys have been discussed in detail in [44]. However, they displayed a similar change tendency. All these results also suggest that there may be different corrosion stages in the corrosion periods of 0–2 h, 2–24 h, and 24–72 h.

### 3.5. EIS Measurements

**Figure 10:**
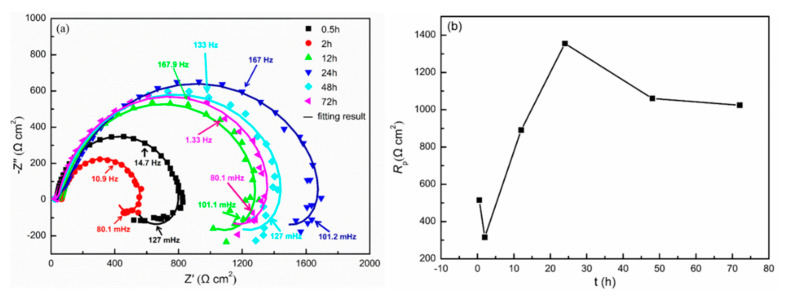
(**a**) Nyquist plots measured at *E*_corr_ for the cast ZK60 alloy immersed in 0.1 M NaCl for different times; (**b**) *R*_p_–t curves at 25 °C.

**Figure 11:**
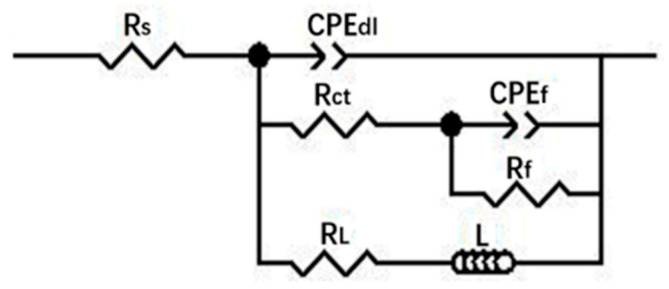
Equivalent circuit used to model the electrochemical impedance spectroscopy (EIS) response of the cast ZK60 alloy in 0.1 M NaCl.

Figure 10 presents the EIS of the cast ZK60 alloy immersed in 0.1 M NaCl for different times and the change in *R*_p_ (polarization resistance) as a function of time. All the Nyquist plots display two capacitive loops in the high-frequency region and an inductive loop in the low-frequency region, which is similar to the EIS features of other Mg alloys [59]. The capacitive loops are related to the processes in the surface film and the electrical double layer [60], and the inductive loop should be attributed to the initiation of the localized corrosion on the surface of the cast ZK60 alloy according to [61,62,63]. Based on these EIS features, Figure 11 presents an equivalent circuit to fit the EIS results in Figure 10a [58,59,64,65]. *R*_s_ is the solution resistance. CPE_f_ and CPE_dl_ represent the constant phase elements (CPE) for the surface film and the electrical double layer, respectively. *R*_f_ and *R*_ct_ represent the surface film resistance and charge-transfer resistance, respectively. *R*_L_ and *L* represent equivalent resistance and inductance to describe the low-frequency inductance. It should be noted that *R*_ct_ is the parallel of the charge-transfer resistance of the anodic process and the cathodic process (*R*_ct,a_ and *R*_ct,c_) at *E*_corr_ [63]. The fitting curves are also displayed in Figure 10a, and the fitting parameters are listed in Table 2.

According to [59,66,67] and the results in Table 2, the fitting curves can be extrapolated to the zero-frequency limit to obtain the polarization resistance (*R*_p_) at different times. Therefore, the *R*_p_–t curve was obtained as shown in Figure 10b. *R*_p_ decreased with time over 0–2 h, indicating corrosion acceleration, and then, *R*_p_ clearly increased between 2 and 24 h before decreasing slightly over 24–72 h. In this case, the change in *R*_p_ with *t* is consistent with that of *P*_i_ (Figure 9b). The *R*_t_ and *R*_f_ values in Table 2 show the same change tendencies as those for *R*_p_. The change in the CPE_dl_, CPE_f_, *R*_L_, and L with *t* should be closely related to the surface layer of the cast ZK60 alloy [59]. The specific changes in these EIS results are discussed later.

### 3.6. Corrosion Morphology of the Cast ZK60 Alloy

**Figure 12:**
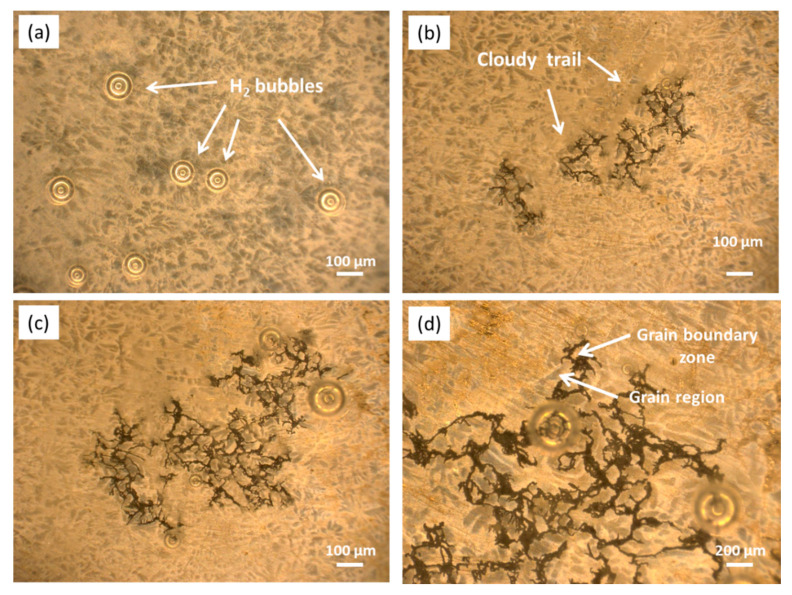
Optical in situ corrosion images of the cast ZK60 etched sample immersed in 0.1 M NaCl for different times: (**a**) 20 min; (**b**) 40 min; (**c**,**d**) 60 min.

To observe the real-time corrosion development in the initial period (0–1 h), Figure 12 presents the in situ corrosion images of the etched sample after immersion in 0.1 M NaCl for different times. In the initial period (20 min, Figure 12a), there was no apparent corrosion, with a limited number of H_2_ bubbles observed on the surface, implying a slight corrosion state. This slight corrosion should be related to the protective film on the alloy surface impeding the corrosion in the initial period [68]. Over 40–60 min (Figure 12b,c), some dark threads appeared on the surface, which lengthened with time and displayed filiform-like corrosion characteristics. In this period, H_2_ bubbles constantly evolved at or near the leading edges of the dark threads, leaving cloudy trails behind them. The magnified picture in Figure 12d suggests that the corrosion filaments seem to traverse within the grain boundary regions and do not extend toward the center of the grains in the initial stage of expansion, which is further verified later. These corrosion characteristics are consistent with those of other Mg alloys in similar corrosion media and should be related to their microstructure features [50,65,69].

**Figure 13:**
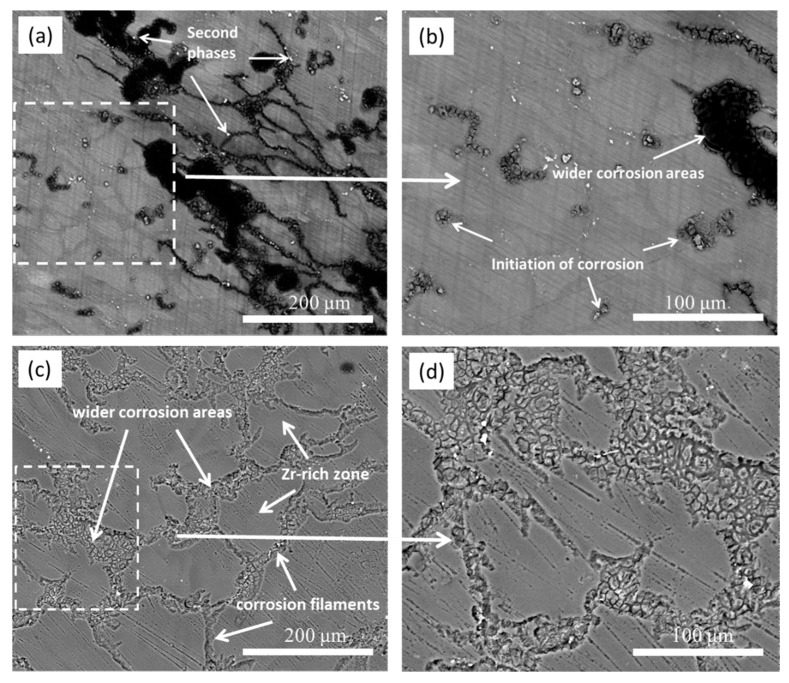
BSE-SEM micrographs of the cast ZK60 etched samples immersed in 0.1 M NaCl for different times: (**a**,**b**) 1 h (with corrosion products); (**c**,**d**) 2 h (without corrosion products).

Figure 13 presents the BSE-SEM micrographs of the etched samples after immersion in 0.1 M NaCl for 1 and 2 h. The black corrosion filaments (Figure 13a) covered by Mg(OH)_2_ [70] mainly occurred on the “darker zones” in grain boundary regions (see Figure 2b and Figure 5) and encompassed the second-phase particles. Some second-phase particles in the boundary regions (Figure 13b) were surrounded by a small number of black corrosion products, implying that the corrosion filaments may have started from the surrounding areas of these second-phase particles. Most of the central regions of the grains, i.e., the Zr-rich areas with a light color (Figure 2b and Figure 5), were still uncorroded. When t = 2 h (Figure 13c), only a small number of second-phase particles occurred in the corroded areas, implying that most of them were removed due to the dissolution of their surrounding Mg matrix. Some broad corrosion areas in Figure 13 show that the corrosion could develop to the central area of grains with increasing corrosion time. Moreover, the corrosion filaments in Figure 13c,d show an apparent corrosion depth, implying that the corrosion also developed under the black corrosion product layer.

**Figure 14:**
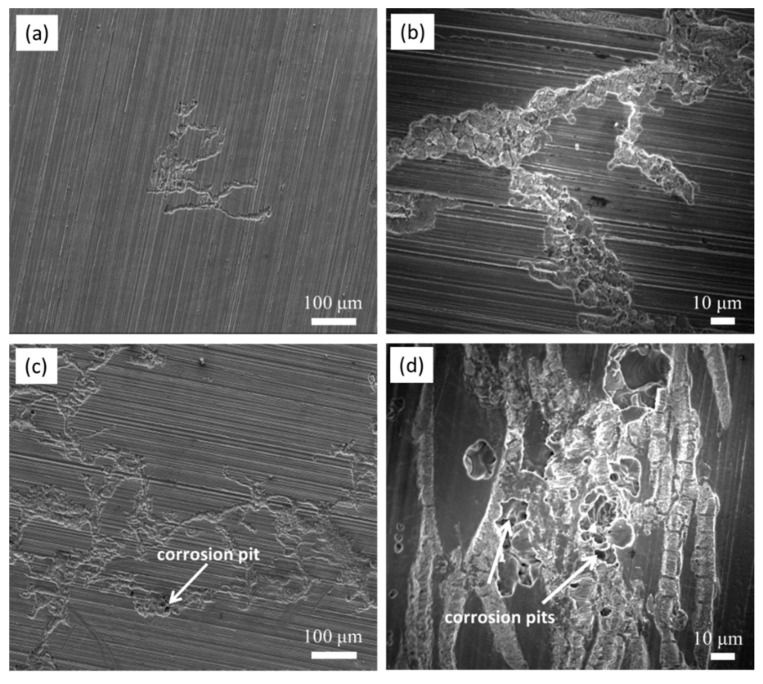
SE-SEM micrographs of the cast ZK60 sample immersed in 0.1 M NaCl for different times: (**a**,**b**) 30 min; (**c**,**d**) 2 h (without corrosion products).

As shown in Figure 12 and Figure 13, the etched samples were used to observe the origination and developmental features of the corrosion filaments on the cast ZK60. To avoid the influence of the etching treatment on the corrosion process, Figure 14 presents the secondary electron (SE) SEM images of the raw alloy sample after immersion in 0.1 M NaCl for 0.5 and 2 h. The corrosion morphologies in Figure 14a,b also exhibit the characteristics of filiform corrosion, similar to those in Figure 12b. The polishing scratches on the surface of the test samples do not appear to influence the initiation and extension of the corrosion filaments, suggesting that they are controlled by the microstructure of the cast ZK60 alloy. When t = 2 h, the corrosion images in Figure 14c,d display features similar to those in Figure 13c and some small corrosion pits occur in the corrosion areas, which may have been the result of the loss of the second phase-particles. The raw cast ZK60 sample also displayed the filiform corrosion features in the early corrosion period (0–2 h).

**Figure 15:**
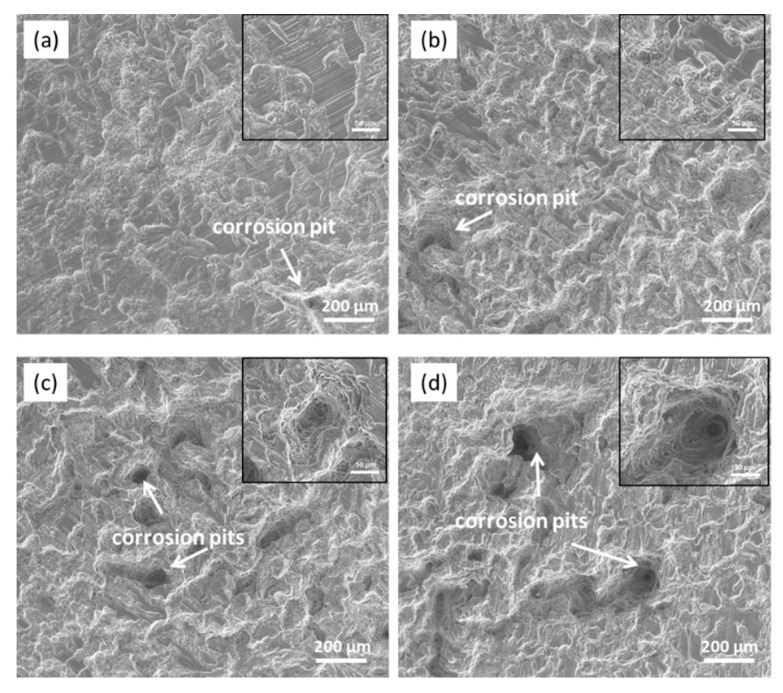
SE-SEM micrographs of the cast ZK60 alloy immersed in 0.1 M NaCl for different times: (**a**) 12 h; (**b**) 24 h; (**c**) 48 h; (**d**) 72 h (with the corrosion products removed).

Figure 15 presents the secondary electron (SE) SEM images of the cast ZK60 alloy after immersion in 0.1 M NaCl for different times to observe the corrosion development over a long period (12–72 h). The corrosion filaments gradually extended to the whole surface of the test samples after 24 h, and the number and depth of the corrosion pits increased with time. These results further prove that with increasing corrosion time, the corrosion of the cast ZK60 alloy can develop toward the central area of grains and toward the depth of the alloy matrix, which is discussed below.

### 3.7. Characterization of the Corrosion Pits on the Cast ZK60 Alloy

**Figure 16:**
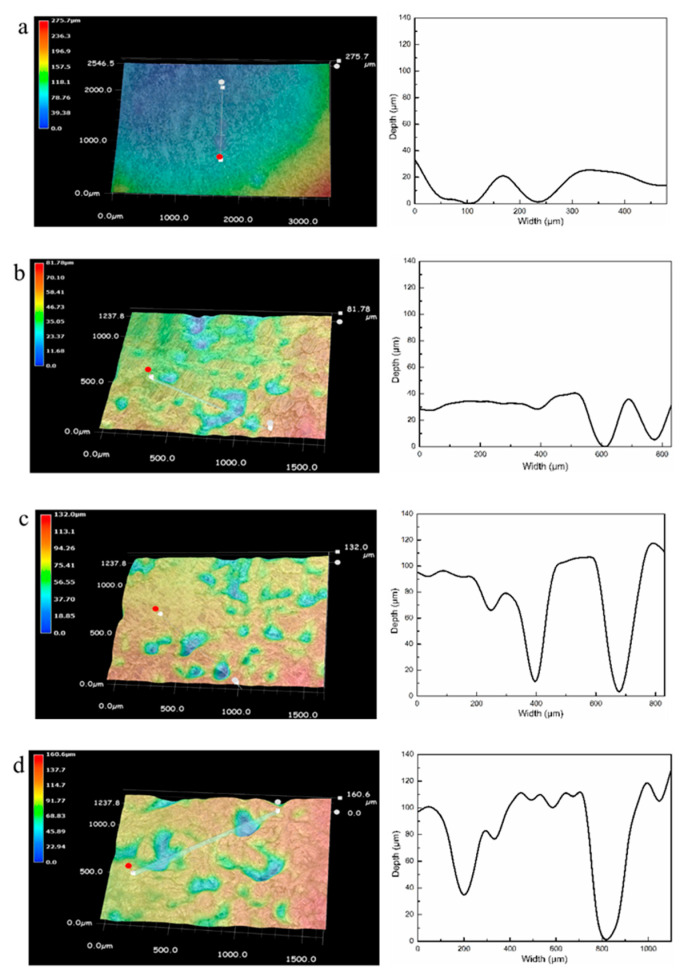
3D corrosion images and depth profiles of the cast ZK60 alloy immersed in 0.1 M NaCl for different times: (**a**) 12 h; (**b**) 24 h; (**c**) 48 h; (**d**) 72 h (without corrosion products).

**Figure 17:**
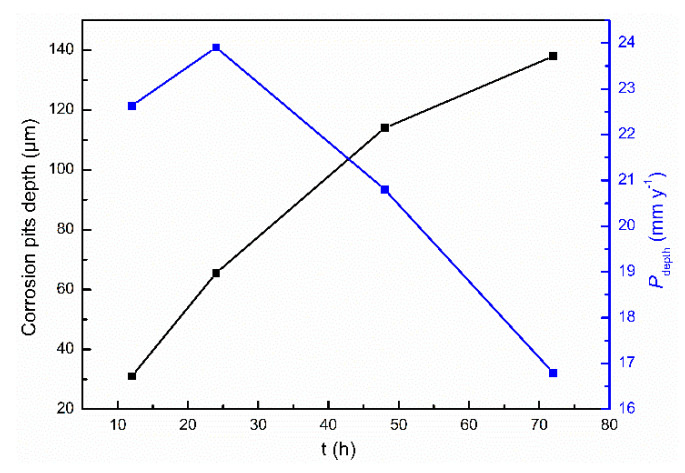
Depth of the deepest corrosion pit and the corresponding *P*_depth_ of the cast ZK60 alloy immersed in 0.1 M NaCl for different times.

To characterize the development of corrosion pits, the deepest pit on the cast ZK60 sample was selected after immersion in 0.1 M NaCl for different times, and their 3D morphologies and depth profiles were measured, as shown in Figure 16. The density and depth of the corrosion pits increased over 12–72 h, and the size of the pit mouth was generally larger than the pit depth, which is similar to the corrosion morphologies in Figure 15. The corresponding pit depth at different corrosion times was employed to calculate the average penetration rate (P_depth_, mm y^−1^), which is also presented in Figure 17. The pit depth increased with time, but the fastest P_depth_ occurred in the period of 12–24 h (~24 mm y^−1^) before gradually decreasing to ~16.6 mm y^−1^ in the period of 24–72 h.

**Figure 18:**
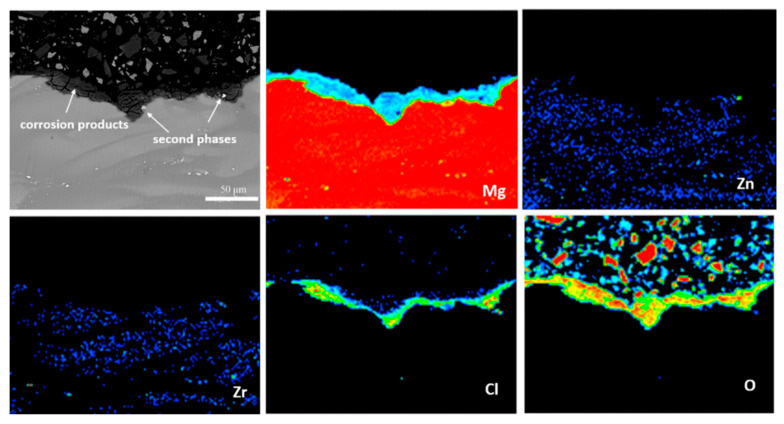
Electron probe micro-analyzer (EPMA) maps of the elemental distribution in the cross-section of a corrosion pit on the cast ZK60 alloy immersed in 0.1 M NaCl for 72 h.

Figure 18 is the cross-sectional EPMA pattern of a corrosion pit on the cast ZK60 sample immersed in 0.1 M NaCl for 72 h. The corrosion pit was covered by the corrosion product, and the second-phase particles were encompassed in it. Only Mg, O, and Cl elements were found in the corrosion product layer, which should result from Mg(OH)_2_ and Cl^−^ in the test solution. Moreover, a large number of Cl^−^ through the whole corrosion product layer suggests that Cl^−^ could penetrate the product layer easily and may become enriched in the bottom of the corrosion pit to propagate the pit corrosion [71]. The second-phase particles in the corrosion pit imply that they may be related to the formation of corrosion pits, which is discussed later.

## 4. Discussion

### 4.1. Initiation and Development of Corrosion on the Cast ZK60 Alloy

#### 4.1.1. Corrosion Reactions on the Cast ZK60 Alloy

A thin oxide film can be formed on the surface of the cast ZK60 alloy in a moist atmosphere at room temperature according to Reactions (5) and (6), which may have a bilayer structure with an inner layer of MgO and an outer layer of Mg(OH)_2_ [72,73].

(5) $$2\mathrm{Mg}+O_{2}=2\mathrm{MgO}$$

(6) $$\mathrm{MgO}+H_{2}O=\mathrm{Mg}{\left(\mathrm{OH}\right)}_{2}$$

Because MgO and Mg(OH)_2_ are both relatively soluble in water according to Reactions (7) and (8) [3,74], where K_sp_ is the solubility product constant, MgO will be gradually dissolved and converted to Mg(OH)_2_ when they are immersed in NaCl solution.

(7) $$\mathrm{MgO}+H_{2}O={\mathrm{Mg}}^{2+}+2{\mathrm{OH}}^{-}\;K_{\mathrm{sp}}=10^{-6}$$

(8) $$\mathrm{Mg}{\left(\mathrm{OH}\right)}_{2}={\mathrm{Mg}}^{2+}+2{\mathrm{OH}}^{-}\;K_{\mathrm{sp}}=10^{-11}$$

In this case, the MgO/Mg(OH)_2_ oxide film formed on the cast ZK60 alloy was partly protective in a neutral NaCl solution. The anodic and cathodic partial reactions of the corrosion process can be written as Reactions (9) and (10), respectively, and the corrosion product is formed as Reaction (11) [75].

(9) $$\mathrm{Mg}={\mathrm{Mg}}^{2+}+2e^{-}$$

(10) $$2H_{2}O+2e^{-}=H_{2}\left(g\right)+2{\mathrm{OH}}^{-}\;$$

(11) $${\mathrm{Mg}}^{2+}+2{\mathrm{OH}}^{-}=\mathrm{Mg}{\left(\mathrm{OH}\right)}_{2}$$

#### 4.1.2. Initiation of Corrosion on the Cast ZK60 Alloy

**Figure 19:**
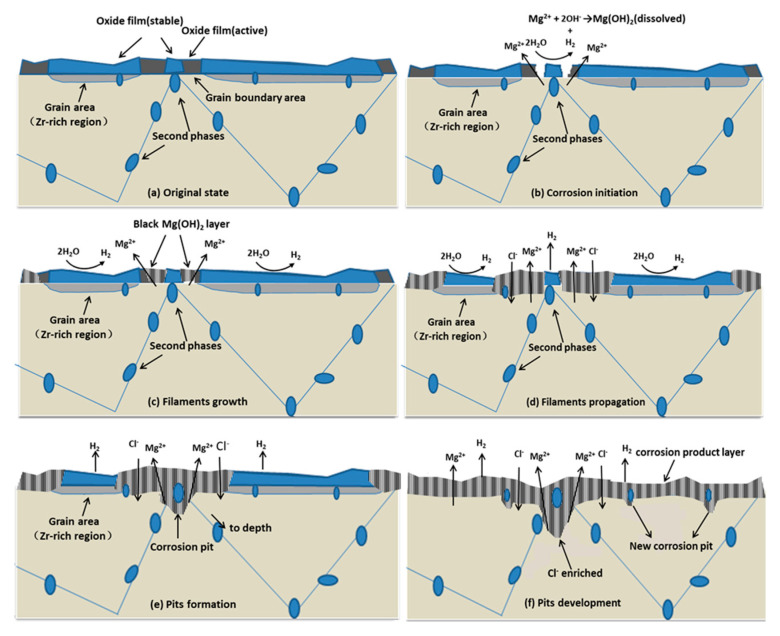
Schematic model for the initiation and development of the corrosion on the cast ZK60 alloy in NaCl solution. (**a**) original state; (**b**) corrosion initiation; (**c**) filaments growth; (**d**) filaments propagation; (**e**) pit formation and (**f**) pit development.

Based on the microstructure of the cast ZK60 alloy, Figure 19 presents a schematic model to illustrate the initiation and development of the corrosion on the cast ZK60 alloy. As discussed above, a partly protective MgO/Mg(OH)_2_ oxide film will be formed on the cast ZK60 alloy in neutral 0.1 M NaCl (Figure 19a). Because the large second-phase particles and the Zr-rich region in the grains were more stable than the Mg matrix in grain boundary regions, as shown in Figure 6, the oxide film in these regions should be more stable than that in their adjacent grain boundary regions (i.e., darker areas in Figure 2b), which was denoted as a stable and active oxide film in Figure 19a [76,77].

Compared to the Mg matrix in the grain boundary regions, those with stable oxide film act as micro-galvanic cathodes. Under the attack of Cl^−^, the film around the second phase could be easily broken due to the higher numbers of imperfections [78]. Therefore, the micro-galvanic corrosion was initiated in the areas around the large discontinuous second-phase particles in the grain boundaries, owing to the strong galvanic effect between these second-phase particles and the Mg matrix in the grain boundaries (Figure 13b). It should be noted that there were some second-phase particles deposited in the grains (Figure 2a and Figure 6), and therefore, the corrosion may also be initiated in the grains. However, we did not observe this phenomenon, which may be due to the stable oxide film in the grains (Zr-rich region) and the light galvanic effect between them.

After the corrosion initiation (Figure 19b), the oxide film near the second-phase particles in the grain boundaries cracked first. However, there were no black Mg(OH)_2_ products observed in this beginning period (Figure 12a), because they were dissolved in the NaCl solution (Equation (5)). After the concentration of Mg(OH)_2_ reached saturation in the NaCl solution, black Mg(OH)_2_ precipitation occurred (Figure 12b).

#### 4.1.3. Growth of Corrosion Filaments on the Cast ZK60 Alloy

Because the Zr-rich area within the grains was more stable than the Mg matrix in grain boundary regions, the corrosion preferentially propagated in the grain boundary regions in the initial corrosion period (Figure 19c), displaying the characteristics of filiform-like corrosion, as shown in Figure 12 and Figure 13. According to [69,70], the front edges of the corrosion filaments acted as intense anodes via Equation (6), while the dark tracks behind the anodes acted as locally activated cathodes, where cathodic Equation (7) occurred. In NaCl solution, Cl^−^ may be enriched around the anode areas to attack and break the oxide film [28]. By contrast, the activated cathodes behind the anodes provide the driving force for the development of the corrosion filaments. Then, the precipitates of Mg(OH)_2_ cover the surface of the active cathodes behind the anodes to make them gradually turn into inert cathodes.

The second phase in the grain boundaries and the Zr-rich region within the grains have an essential influence on the origination and propagation of the corrosion filaments on the cast ZK60. These filiform-like corrosion characteristics of the cast ZK60 were quite different from those of Mg-3Zn and Mg-8Li alloys reported previously [65,71], in which the corrosion filaments seem not to be affected by the second phases and can originate from the center of the grains and spread to various directions. These differences should be ascribed to their different microstructures, especially the quantity and size of the second phase and the distribution of alloying elements in these alloys.

As the corrosion time was increased, the active “dark areas” along the grain boundaries were gradually covered with a black Mg(OH)_2_ layer (Figure 19c) and potentially became cathodic areas [79]. This change would impede the anodic corrosion process in these areas and make their potential positive, reducing and even eliminating the potential difference between these areas and the Zr-rich regions. Therefore, the corrosion filaments could gradually develop to the central area of grains (Figure 19d) to form some broad corrosion areas, as shown in Figure 13 and Figure 14. Additionlly, the black Mg(OH)_2_ layer was porous and imperfect and could not prevent the entry of the NaCl solution (Figure 18). Thus, the anodic process still existed beneath the Mg(OH)_2_ layer, developing an apparent corrosion depth under the corrosion filaments, as shown in Figure 13 and Figure 14. When t > 24 h, most of the alloy surface was gradually covered with the Mg(OH)_2_ layer (Figure 15), and finally, it would be covered completely (Figure 19e,f).

#### 4.1.4. Development of Corrosion Pits on the Cast ZK60 Alloy

With the development of the corrosion filaments, the corrosion pits will be formed when the Mg matrix surrounding the large second-phase particles is corroded completely (Figure 19e). Some small corrosion pits occurred in the corrosion filaments when t = 2 h (Figure 14c,d), which may be due to the loss of the second-phase particles when the corrosion product was removed. The visible corrosion pits occurred after 12 h of immersion (Figure 15), and their number and depth increased with time over 24–72 h, in which P_depth_ was about one magnitude order higher than those corrosion rates (P_w_, P_H_, and P_i_), as shown in Figure 15, Figure 16 and Figure 17. The high P_depth_ of the cast ZK60 alloy indicates a severe localized corrosion state and warrants further investigation.

The development of the corrosion pits was related to the occluded environment under the corrosion filaments, covered by the second-phase particles and Mg(OH)_2_ precipitate. The solution in the occluded corrosion pit would be alkalized to increase the pH to around 10.5 through Reaction (5). At the same time, Cl^−^ would migrate in due to the charge neutralization and become enriched at the bottom of the pit (Figure 18). These effects would accelerate the anodic process in the corrosion pits and make them develop deeply [80].

According to the above discussion, the microstructure of the cast ZK60 alloy had an essential influence on the corrosion initiation and propagation in NaCl solution. Thus, it is necessary to improve its corrosion resistance by microstructure modification, which will be studied later.

### 4.2. Change in the Corrosion Rate in Different Corrosion Periods

The P_H_, P_i_, and R_p_ values of the cast ZK60 alloy generally showed different change tendencies in different immersion periods (Figure 8, Figure 9b and Figure 10b). In the early corrosion period (0–2 h), the corrosion rates (P_H_ and P_i_) increased with time (Figure 8b and Figure 9b), while R_t_, R_f_, and R_p_ decreased (Table 2 and Figure 10b). In this period, the filiform corrosion was initiated and developed quickly on the cast ZK60 alloy without visible corrosion pits (Figure 12, Figure 13 and Figure 14), corresponding to the states in Figure 19a–d. In this case, with the development of the corrosion filaments, the broken oxide film increased steadily to accelerate the anodic and cathodic processes, as shown in Figure 9, and made the corrosion rates increase. The increase in the broken oxide film may be the reason why no E_break_ occurred in the anodic polarization curve when t = 2 h (Figure 9). Furthermore, the increase in the broken oxide film enlarged the reactive areas on the cast ZK60 surface to increase CPE_dl_ and CPE_f_, meanwhile decreasing R_L_ and L in this period (0–2 h, Table 2).

The second corrosion period was determined to be within 2–24 h, according to the above discussion about the corrosion development on the cast ZK60 alloy. In this period, the corrosion filaments gradually occupied most of the active oxide film, and a small number of corrosion pits occurred and developed quickly (Figure 15 and Figure 16), which corresponds to the state in Figure 19e. Because most of the active oxide film was corroded and covered with a black Mg(OH)_2_ precipitate, the anodic process, Reaction (9), was inhibited significantly to make E_corr_ more positive, and E_break_ occurs again with a more positive value (Figure 9). In this case, the corrosion rate (P_H_ and P_i_) decreased significantly (Figure 8b and Figure 9b), while R_t_, R_f_, and R_p_ increased (Table 2 and Figure 10b). The difference is that P_i_ and 1/R_p_ reached the lowest value at t = 24 h, but P_H_ decreased persistently until t = 48 h, while P_w_ as shown in Figure 7 displayed the highest value at t = 24 h, which may be ascribed to the differences among these test methods. In this period, the decrease in the broken oxide film may also result in a clear increase in R_L_ and L and decrease in CPE_dl_ (Table 2). Meanwhile, CPE_f_ firstly increased and then decreased, which may have been due to the increase in the area and thickness of the corrosion product layer. Even though very few corrosion pits occurred in this period (Figure 15 and Figure 16), their P_depth_ increased significantly and reached a large value of about 24 mm y^−1^ (Figure 17), which was much larger than the general corrosion rates of P_w_, P_H_, and P_i_ in this period (Figure 7, Figure 8 and Figure 9b). Therefore, the pitting corrosion became more critical than the filiform corrosion in this period.

Over 24–72 h, most of the surface of the cast ZK60 was gradually covered with Mg(OH)_2_ layer, but the number and depth of the corrosion pits increased (Figure 15, Figure 16 and Figure 17), corresponding to the state in Figure 19f. Because of the increase in corrosion pits, the anodic process was accelerated again. In this case, the E_break_ and E_corr_ of the cast ZK60 alloy became negative, and P_i_ increased again (Figure 9), while R_t_, R_f_, and R_p_ decreased (Table 2 and Figure 10b). However, P_w_ and P_depth_ decreased with time in this period (Figure 7 and Figure 17), indicating that the increase in the corrosion product layer still impeded the growth rate of the pitting depth and the general weight loss, possibly due to the alkalizing effect of the cathodic reaction and the low solubility of Mg(OH)_2_. In this period, the increase in CPE_f_ may have been due to the dissolution of the corrosion product layer reducing its thickness; meanwhile, the decrease in R_L_ and L may have been related to the acceleration of the pitting corrosion process, similar to in the processes on passive metals [61]. In general, pitting corrosion became more severe in this period and dominated the corrosion behavior of the cast ZK60 alloy. In this case, P_depth_ should be adequate to describe the corrosion rate of the cast ZK60 alloy in this period.

## 5. Conclusions

1. The microstructure of the cast ZK60 alloy was composed of an α-Mg phase and large second-phase particles (MgZn_2_), which mainly deposited along the grain boundaries, and a Zr-rich region existed in the central area of the grains. The grain boundaries and their adjacent regions (noted as grain boundary regions) had relatively higher Mg contents.
2. The second-phase particles and the central area of the grains (Zr-rich region) were more electrochemically stable than the grain boundary regions, resulting in a strong micro-galvanic effect between them.
3. The corrosion of the cast ZK60 alloy in 0.1 M NaCl solution originated from the areas around the second-phase particles in grain boundaries and firstly developed in the grain boundary regions, showing filiform-like corrosion characteristics owing to the strong micro-galvanic effect in its microstructure.
4. The general corrosion rate increased in the early corrosion period (about 0–2 h). Then, the black corrosion filaments covered with Mg(OH)_2_ gradually occupied most of the alloy surface to inhibit the corrosion process and decrease its general corrosion rate. However, corrosion pits occurred under the corrosion filaments and had a high growth rate (P_depth_) in this period. After 24 h, the number of corrosion pits increased, and P_depth_ decreased with time (17–24 mm y^−1^) but was still eight times larger than the general corrosion rates (P_w_, P_H_, and P_i_), which should be paid more attention. The microstructure of the cast ZK60 alloy has an essential influence on the initiation and development of its corrosion.

## Figures and Tables

**Table 1:** Chemical composition of the cast ZK60 alloy (wt.%).

Zn	Al	Fe	Ni	Cu	Zr	Mg
5.80	<0.01	<0.01	<0.01	<0.01	0.57	Bal.

**Table 2:** Fitting parameters of the EIS results in Figure 9a.

Time h	*R*_s_ Ω·cm^2^	*R*_ct_ Ω·cm^2^	CPE_dl_-T μFcm^−2^Hz^1^^−n1^	CPE_dl_-P n1	*R*_f_ Ω·cm^2^	CPE_f_-T μFcm^−2^Hz^1^^−n2^	CPE_f_-P n2	*R*_L_ Ω·cm^2^	*L* H·cm^2^	Chi-Squared Error
0.5	32	26	22	0.87	759	16	0.69	1360	2359	1.55 × 10^−2^
2	65	14	67	0.87	502	21	0.96	1224	1143	1.02 × 10^−2^
12	47	37	26	0.85	1254	50	0.89	2686	8005	1.33 × 10^−2^
24	40	179	38	0.79	1546	22	0.88	5865	16,588	1.23 × 10^−2^
48	27	58	37	0.81	1453	48	0.87	3429	9781	1.48 × 10^−2^
72	28	34	38	0.83	1390	59	0.88	3557	9029	1.67 × 10^−2^
